# The review of the genus *Coccinella* (Coleoptera, Coccinellidae) from Pakistan

**DOI:** 10.3897/BDJ.12.e137417

**Published:** 2024-11-21

**Authors:** Zafar Iqbal, Rashid Azad, Xu Jin, Muhammad Asghar Hassan, Munawar Abbas, Muhammad Farooq Nasir, Imran Bodlah, Muhammad Ali, Karol Szawaryn, Rui-E Nie

**Affiliations:** 1 The Anhui Provincial Key Laboratory of Biodiversity Conservation and Ecological Security in the Yangtze River Basin, College of Life Sciences, Anhui Normal University, Wuhu, 241000 Anhui, China The Anhui Provincial Key Laboratory of Biodiversity Conservation and Ecological Security in the Yangtze River Basin, College of Life Sciences, Anhui Normal University Wuhu, 241000 Anhui China; 2 Department of Entomology, The University of Haripur, Haripur, Khyber Pakhtunkhwa, Pakistan Department of Entomology, The University of Haripur Haripur, Khyber Pakhtunkhwa Pakistan; 3 The Anhui Provincial Key Laboratory of Biodiversity Conservation and Ecological Security in the Yangtze River Basin, College of Life Sciences, Anhui Normal University, Wuhu, Anhui, 241000, China The Anhui Provincial Key Laboratory of Biodiversity Conservation and Ecological Security in the Yangtze River Basin, College of Life Sciences, Anhui Normal University Wuhu, Anhui, 241000 China; 4 Institute of Entomology, The Provincial Special Key Laboratory for Development and Utilization of Insect Resources, Guizhou University, Guiyang 550025, China Institute of Entomology, The Provincial Special Key Laboratory for Development and Utilization of Insect Resources, Guizhou University Guiyang 550025 China; 5 College of Food Science and Technology, Henan University of Technology, Zhengzhou, Henan, 450001, China College of Food Science and Technology, Henan University of Technology Zhengzhou, Henan, 450001 China; 6 Department of Entomology, Faculty of Crop and Food Sciences, Pir Mehr Ali Shah Arid Agriculture University, Rawalpindi, Pakistan Department of Entomology, Faculty of Crop and Food Sciences, Pir Mehr Ali Shah Arid Agriculture University Rawalpindi Pakistan; 7 Department of Zoology, Faculty of Natural and Health Sciences, University of Baltistan, Skardu, Pakistan Department of Zoology, Faculty of Natural and Health Sciences, University of Baltistan Skardu Pakistan; 8 Museum and Institute of Zoology Polish Academy of Science, Twarda 51/55, 00-818 Warsaw, Poland Museum and Institute of Zoology Polish Academy of Science Twarda 51/55, 00-818 Warsaw Poland; 9 ‡ The Anhui Provincial Key Laboratory of Biodiversity Conservation and Ecological Security in the Yangtze River Basin, College of Life Sciences, Anhui Normal University, Wuhu, 241000 Anhui, China ‡ The Anhui Provincial Key Laboratory of Biodiversity Conservation and Ecological Security in the Yangtze River Basin, College of Life Sciences, Anhui Normal University Wuhu, 241000 Anhui China

**Keywords:** Asia, barcoding, Coccinelloidea, COI, new record, Oriental Region

## Abstract

**Background:**

The genus *Coccinella* is reviewed with seven species found in Pakistan: *C.luteopicta* (Mulsant, 1866), *C.marussii* Kapur, 1973, *C.iranica* Dobzhansky, 1926, *C.transversalis* Fabricius, 1781, *C.septempunctata* Linnaeus, 1758, *C.transversoguttatatransversoguttata* Faldermann, 1835 and *C.undecimpunctata* Linnaeus, 1758. Information on prey, host plants, distribution and an identification key for *Coccinella* species in Pakistan is provided. Additionally, newly-sequenced partial COI (cytochrome-c-oxidase subunit I) for *C.luteopicta* and *C.marussii* were used to determine their phylogenetic positions within the genus *Coccinella*.

**New information:**

This study comprehensively reviews the genus *Coccinella* in Pakistan and highlights *Coccinellaluteopicta* as a new country record. Morphological features of adults, including male genital characters and an identification key to known species in Pakistan are presented. Records of prey, host plants and distributions for all identified species are included. The new data (COI-barcode) shows that *C.luteopicta* (Mulsant, 1866) was recorded first in Pakistan.

## Introduction

The family Coccinellidae (Coleoptera) comprises over 6,000 species worldwide (Vandenberg 2002, Ślipiński 2007, Robertson et al. 2015) and is currently divided into three subfamilies, Coccinellinae, Monocoryninae and Microweiseinae (Che et al. 2021).

The genus *Coccinella* belongs to the tribe Coccinellini within the subfamily Coccinellinae. It was established by Linnaeus in 1758. Subsequently, Latreille (1807) designated *Coccinella* as the type genus of the family Coccinellidae. Dobzhansky (1925) established the foundations of the modern classification of the genus *Coccinella*. He was the first one to precisely define key characters of the genus, using a distinction in the colour pattern and particularly in the structure of male and female genitalia to separate the genera *Oenopia* Mulsant, 1850 (= *Synharmonia* Ganglbauer, 1899) and *Coccinula* Dobzhansky, 1925. Later, he examined 12 Palaearctic (Dobzhansky 1926) and 10 Nearctic species (Dobzhansky 1931) of *Coccinella* based on the characters of male and female genitalia. Iablokoff-Khnzorian (1979) catalogued 26 Palaearctic species of *Coccinella* using type material. Kovář (2005) revised the Palaearctic species of the *C.transversoguttata* species group. Regional catalogues of Palaearctic and Oriental species were published by Jacobson (1915), Poorani (2002), Kovář (2005), Kovář (2007) and Poorani (2023). This genus contains about 50 species primarily occurring in the Holarctic Region (Ślipiński et al. 2020) extending to Africa, the Orient and the Pacific (Poorani 2023).

Members of this genus are aphidophagous (Ali et al. 2018) and play a vital role in regulating the population of a wide range of soft-sucking insect pests, including aphids, adelgids, psyllids, whiteflies, scale insects and mealybugs (Rafi et al. 2005). To date, six species of *Coccinella* have been reported from Pakistan (Ahmad 1968, Hashmi and Tashfeen 1992, Khan et al. 1999, Rafi et al. 2005, Ullah et al. 2012, Ali et al. 2012, Ashfaque et al. 2013, Hayat et al. 2017, Iqbal et al. 2017, Ali et al. 2018, Iqbal et al. 2020). The current study summarises the information about *Coccinella* species in Pakistan including *C.luteopicta* as a new country record. The prey species, associated plants, geographical distribution and an identification key to the genus *Coccinella* species in Pakistan are provided. Additionally, cytochrome-c-oxidase subunit I (COI) DNA sequence data of *C.luteopicta* and *C.marussii* were generated for the first time to double-check their position.

## Materials and methods

**Specimens Collection and identification**

The adult specimens of ladybird beetles, prey and associated plants were collected from various localities in northern Pakistan during 2015 and 2022. Collected beetles were preserved in 95% ethanol and male genitalia were dissected and cleared in 10% potassium hydroxide (KOH). Specimens were examined and photographed using a Nikon digital camera attached to stereomicroscope SMZ1500. Furthermore, specimens were identified using the following publications: Ahmed et al. (2017), Sasaji (1968), Sasaji (1971), Canepari (1997), Kuznetsov (1997), Kovář (2005), Rafi et al. (2005), Ali et al. (2018) and Poorani (2023). We followed Che et al. (2021) for taxonomic classification; morphological terminology follows Ślipiński (2007).

The following body measurements were taken using an ocular micrometer: total length (**TL**): from the tip of the clypeus margin to the tip of the elytra; total width (**TW**): across the widest part of both elytra; head width (**HW**): in a frontal view; total height (**TH**): across the highest point of the elytra: pronotal width (**PW**): at widest part; pronotal length (**PL**): from the middle of the base to anterior margin of pronotum; elytral length (**EL**): from apex to base including scutellar shield.

Photographs were edited using Adobe Photoshop CS6 and Helicon Focus 8.1.0. Specimens are deposited at the Laboratory of Biosystematics, Department of Entomology, Pir Mehr Ali Shah Arid Agriculture University Rawalpindi, Pakistan (**PMAS-AAUR**) and the National Insect Museum, National Agriculture Research Center, Islamabad (**NIM**).

**DNA Barcoding sequencing and analysis**

DNA was extracted from the thoracic muscles of the selected adult specimen using the DNeasy Blood and Tissue Kit (Qiagen, Germany) following the manufacturer’s protocol. Amplification targeting a 418 base pair region of the mitochondrial cytochrome oxidase subunit I (COI) gene with invertebrate-specific primers: Primer_F (CCNGAYATRGCNTTYCCNCG) and Primer_R (TANACYTCNGGRTGNCCRAARAAYCA) (Folmer et al. 1994, Arribas et al. 2016). Polymerase chain reaction (PCR) was prepared in a volume of 25 μl, containing 17.9 μl of PCR water, 2.5 μl of buffer, 0.75 μl of MgCl_2_, 0.75 μl of dNTP, 0.75 μl of each primer and 2 μl of template DNA. PCR conditions included an initial denaturation step of 4 min at 94ºC; 35-40 cycles with denaturation for 30 sec at 94ºC, an annealing step for 30 sec at 48ºC and an extension step for 45 sec at 72ºC; and a final extension step of 10 min at 72ºC. The primer with different tags of the PCR is 6 bp. Amplicon pools were cleaned using AMPure XP magnetic beads and used as templates for a limited-cycle secondary PCR amplification to add dual-index barcodes and the Illumina sequencing adapters (Nextera XT Index Kit; Illumina). The resulting barcoding libraries were sequenced on an Illumina MiSeq sequencer (2 × 300 bp paired-end reads) on ~ 1% of each of the flow cells and HiSeq 2500 (2 × 250 bp paired-end reads). Sequence processing was carried out by an established pipeline for quality filtering, trimming and merging (Creedy et al. 2019). New barcode data were deposited in GenBank under accession numbers: PP853180 and PP853181 (Suppl. material 1). To construct the phylogenetic tree, we downloaded 23 COI sequences from GenBank and included sequences of *Coccinellaseptempunctata* (PP066015) and *Coccinellatransversoguttatatransversoguttata* (PP066016) from specimens collected in Pakistan. Our analyses included 15 taxa of the genus *Coccinella*; *Afidentulamanderstjernae* (PP066012) and *Henosepilachnavigintioctopunctata* (PP066013) were treated as outgroups (Suppl. material 1).

TransAlign (Bininda-Emonds 2005) was used to prepare sequence alignment. IQ-TREE programme (Minh et al. 2020) was used for Maximum Likelihood calculations with 1000 ultrafast bootstraps under model (TIM2+R3+F) selected using ModelFinder (Nguyen et al. 2015), implemented in the IQ-TREE programme. For the Bayesian Inference, MrBayes v.3.2.7a (Ronquist et al. 2012) has been used with two parallel runs and 20,000 generations from which the initial 25% of sampled data were discarded as burn-in. Substitution model for the BI analysis (GTR+I+G+F) was selected using PartitionFinder v.2.1.1 (Lanfear et al. 2017).

## Taxon treatments

### Coccinella

Linnaeus, 1758

DF6BEC79-AD09-55E7-8C97-9CDC15035391

Coccinella Linnaeus, 1758: 364. Type species: *Coccinellaseptempunctata* Linnaeus, 1758, subsequently designated by Latreille (1810): 432.

#### Diagnosis

**Figure 1.**
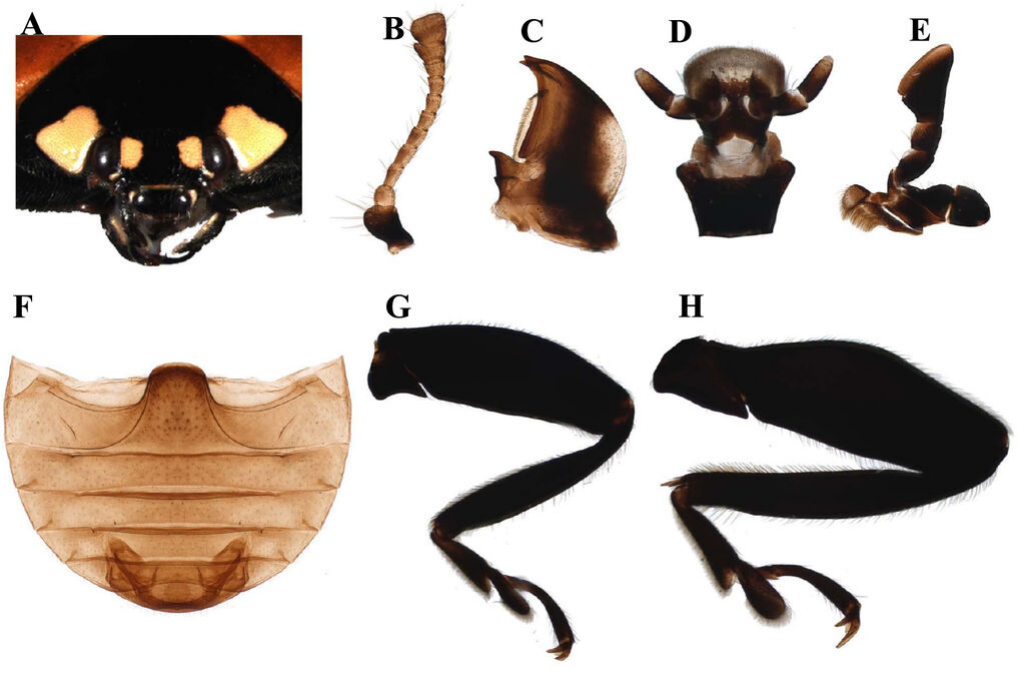
Elements of morphology of the genus *Coccinella* Linnaeus using *Coccinellaseptempunctata* Linnaeus. **A** Head; **B** Antenna; **C** Mandible; **D** Labium; **E** Maxilla; **F** Fore-leg; **G** Hind-leg.

*Coccinella* (Fig. 1A-H) can be distinguished from other genera by the following characters: body 4.5 to 8.5 mm in length, weakly convex and dorsal surface glabrous; anterior clypeal margin straight, with projections at lateral margins; antennae (Fig. 1B) composed of 11 antennomeres, shorter than head capsule, antennal club composed of 3 antennomeres, terminal antennomere apically round or truncate; terminal maxillary palpomere (Fig. 1E) strongly securiform; abdominal postcoxal line laterally incomplete and with additional oblique dividing line; tibiae of legs (Figs. 1G-H) with two spurs at apex; tarsal claw with large subquadrate basal tooth.

### Coccinella luteopicta

(Mulsant, 1866)

7C0914A5-A5D7-54AD-BA31-6CC29B0BBF0F

Adalia luteopicta Mulsant, 1866: 45; Korschefsky (1932): 433.
Lioadalia luteopicta : Crotch (1874): 104; Mader (1930): 134; Kapur (1958): 326; Miyatake (1967): 74; Bielawski (1971): 7; Kapur (1973): 32; Gordon (1987): 12.
Coccinella luteopicta : Iablokoff-Khnzorian (1982): 395; Canepari (1997): 52; Poorani (2002): 325; Ren et al. (2009): 186; Poorani (2023): 81.

#### Materials

- **Type status:** Other material. **Occurrence:** individualCount: 1; sex: 1 male; occurrenceID: 386FCE80-95B9-520E-8BC3-85AB6F23CE44; **Taxon:** scientificName: *Coccinellaluteopicta*; order: Coleoptera; family: Coccinellidae; scientificNameAuthorship: Mulsant, 1866; **Location:** country: Pakisan; stateProvince: Gilgit-Baltistan; locality: Ganche, Barah; verbatimElevation: 2466 m; decimalLatitude: 35.20556; decimalLongitude: 76.27444; **Event:** samplingProtocol: mist net; year: 2015; month: 7; day: 13; **Record Level:** institutionCode: PMAS-AAUR
- **Type status:** Other material. **Occurrence:** individualCount: 5; sex: 2 males, 3 females; occurrenceID: 25BD8176-74F1-5D9D-8205-085080CF1A18; **Taxon:** scientificName: *Coccinellaluteopicta*; order: Coleoptera; family: Coccinellidae; scientificNameAuthorship: Mulsant, 1866; **Location:** country: Pakisan; stateProvince: Gilgit-Baltistan; locality: Shigar, Chu-Tran; verbatimElevation: 2434 m; decimalLatitude: 35.70189; decimalLongitude: 75.40967; **Event:** samplingProtocol: mist net; year: 2017; month: 8; day: 27; **Record Level:** institutionID: PMAS-AAUR
- **Type status:** Other material. **Occurrence:** individualCount: 4; sex: 2 males, 2 females; occurrenceID: 8DC5F3A3-18E2-58EF-BFA2-BB8155EBFB74; **Taxon:** scientificName: *Coccinellaluteopicta*; order: Coleoptera; family: Coccinellidae; scientificNameAuthorship: Mulsant, 1866; **Location:** country: Pakisan; stateProvince: Gilgit-Baltistan; locality: Ganche, Barah; verbatimElevation: 2466 m; decimalLatitude: 35.20556; geodeticDatum: 76.27444; **Event:** samplingProtocol: mist net; year: 2021; month: 8; day: 1; **Record Level:** institutionID: PMAS-AAUR

#### Description

**Figure 2.**
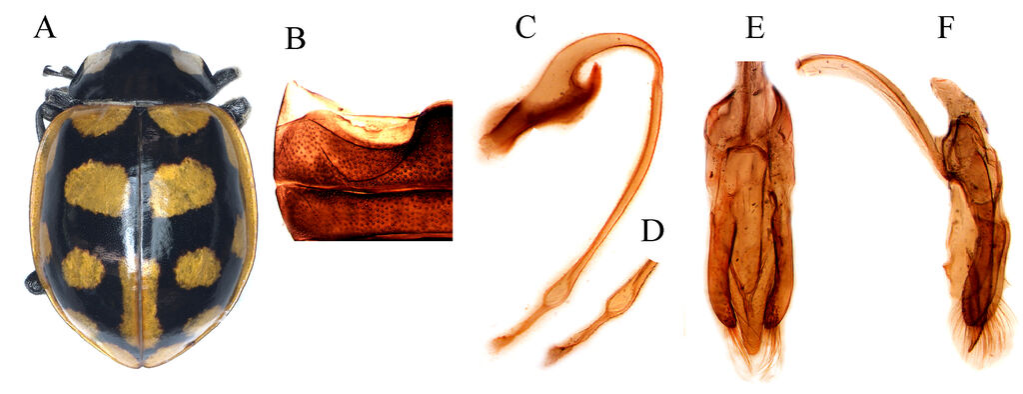
*Coccinellaluteopicta* (Mulsant). **A** dorsal view; **B** abdomen; **C** penis; **D** penis apex; **E** tegmen inner view; **F** tegmen lateral view.

Body (Fig. 2A) elongate-oval and convex, dorsal surface glabrous; head black, mouth-parts and antennae dark-brown; pronotum black with white sub-quadrated marks at antero-lateral margins; scutellar shield completely black; elytra bright red, with reticulate black pattern; ventral side and legs black; prosternal process narrow and carinae present; abdominal postcoxal line (Fig. 2B) recurved and incomplete, expending 1/4 length of ventrite 1 and with indistinct oblique dividing line. Male genitalia (Fig. 2C-F) with penis apex flattened, slightly swollen at 7/9 of its length and then narrowing towards the blunt tip (Fig. 2D), parameres somewhat shorter than the penis guide, with long hairs at inner sides and apices. Penis guide in inner view (Fig. 2E) broader basally, 3/5 length parallel sides, gradually tapering towards the blunt tip.

##### Measurements

TL: 6.20–6.37 mm; TW: 4.27–4.36 mm; HW: 1.40–1.44 mm; TH: 2.01–2.03 mm; EL/TW: 1.10-1.15; TL/TW: 1.46; PL/PW: 0.52-0.54; PW/EW: 0.65-0.66.

#### Diagnosis

*Coccinellaluteopicta* closely resembles *C.transversalis* and *C.marussii* in general appearance, but it can easily be identified by having a reticulate black pattern on the elytra, elytra suture with anterior half-black, penis guide in inner view broader at the base and then gradually tapering towards the blunt tip (Fig. 2E).

#### Distribution

Pakistan: Gilgit-Baltistan; – India, Nepal, China (Tibet, Yunnan) (Canepari 1997, Poorani 2002, Kovář 2007, Poorani 2023). **New country record.**

#### Ecology

**Prey.** In the present study, *C.luteopicta* was feeding on *Therioaphistrifolii* (Monell) and *Brevicorynebrassicae* (Linnaeus) (Hemiptera, Aphididae).

**Associated Plants.** This species was collected from *Trifoliumalexandrinum* Linnaeus (Fabales, Fabaceae) and *Brassicaoleracea* Linnaeus (Brassicales, Brassicaceae) in this study.

### Coccinella marussii

Kapur, 1973

0425A937-E571-5422-A455-B0637D2E2602

Coccinella marussii Kapur, 1973: 374; Poorani (2002): 326; Kovář (2005): 132; Poorani (2023): 82.

#### Materials

- **Type status:** Other material. **Occurrence:** individualCount: 7; sex: 3 males, 4 females; occurrenceID: 39E28467-F793-5EEB-AB98-AA1015BA1340; **Taxon:** scientificName: *Coccinellamarussii*; order: Coleoptera; family: Coccinellidae; scientificNameAuthorship: Kapur, 1973; **Location:** country: Pakistan; stateProvince: Gilgit-Baltistan; locality: Ghizer, Phandar (Langhar); verbatimElevation: 3551 m; decimalLatitude: 36.04033; decimalLongitude: 72.63111; **Event:** year: 2017; month: 6; day: 30; **Record Level:** institutionCode: PMAS-AAUR

#### Description

**Figure 3.**
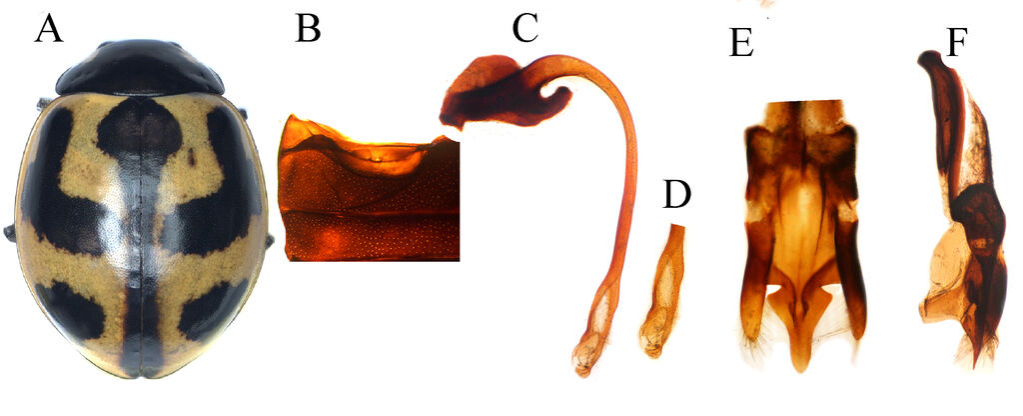
*Coccinellamarussii* Kapur: **A** dorsal view; **B** abdomen; **C** penis; **D** penis-apex; **E** tegmen inner view; **F** tegmen lateral view.

Body (Fig. 3A) oval and slightly convex, dorsally glabrous; head almost black with triangular pale-yellow spots on each side of frons close to eyes, but not touching eye margin; mouthparts and antennae black or dark-brown; pronotum black with pale yellow quadrangular marking at anterior corners; scutellar shield black; each elytron in nominative form yellow-orange, with five black spots on each elytron, in examined specimens, three antero-lateral spots (humeral, lateral, middle) merged, forming L-shape in anterior portion of elytron; apical spot large sub-oval; scutellar macula large, subcordate; elytral suture with the broad black band; ventral side and legs black, except prothoracic hypomeron pale yellow; elytral epipleura yellow-orange; abdominal postcoxal lines (Fig. 3B) laterally incomplete, descending and expanding to posterior margin of ventrite 1, with oblique dividing line distinct, touching or close to the apex of postcoxal line. Male genitalia can be seen in Fig. 3C-F.

##### Measurements

TL: 5.23–5.64 mm; TW: 3.89–4.09 mm; HW: 1.35–1.43 mm; TH: 1.95–2.34 mm; EL/TW: 1.05; TL/TW: 1.35–1.38; PL/PW: 0.54–0.57.

#### Diagnosis

This species is similar to *C.luteopicta* and *C.transversalis* in external appearance, but it can be distinguished by the apex of the penis guide with a small hastate in the inner view (Fig. 3E) and tongue-shaped apex in the lateral view (Fig. 3F).

#### Distribution

Pakistan: Gilgit-Baltistan (Kovář 2005, current study); – India (Kashmir) (Kapur 1973, Kovář 2005), Turkey (Fürsch 1981).

#### Ecology

**Prey.** In the present study, *C.marussii* predates on *Macrosiphum* sp. (Hemiptera, Aphididae).

**Associated Plants.** This species was collected from *Rosawebbiana* Wallich ex Royle (Rosales) and *Ribes* sp. (Saxifragales, Grossulariaceae) (current study).

#### Notes

This is a rare species in the family Coccinellidae with limited distribution (Kapur 1973, Kovář 2005). See Kovář (2005) for a detailed description and illustration. Poorani (2023) also described and illustrated it from India.

### Coccinella transversalis

Fabricius, 1781

D3E05E1A-5BAF-5A97-B5A5-CAAA83A177D0

Coccinella transversalis Fabricius, 1781: 97; Mulsant (1850): 126; Timberlake (1943): 14; Iablokoff-Khnzorian (1979): 68; Iablokoff-Khnzorian (1982): 391; Pope (1989): 652; Poorani (2002): 326; Rafi et al. (2005): 46; Ali et al. (2012): 170. Abdolahi et al. (2018): 14; Ślipiński et al. (2020): 33; Poorani (2023): 88.
Coccinella repanda : *Thunberg (1781)*: 18; Mulsant (1850): 1022; Crotch (1871): 3; Crotch (1874): 11; Korschefsky (1932): 423; Pope (1987): 62; Ślipiński et al. (2020): 33.
Coccinella contempta : Boisduval (1835): 59; Mulsant (1850): 1022; Pope (1989): 653; Ślipiński et al. (2020): 33.

#### Materials

- **Type status:** Other material. **Occurrence:** individualCount: 2; sex: 1 male, 1 female; occurrenceID: 99975340-D8BD-5D6D-A341-B8428C0B9062; **Taxon:** scientificName: *Coccinellatransversalis*; order: Coleoptera; family: Coccinellidae; scientificNameAuthorship: Fabricius, 1781; **Location:** country: Pakistan; stateProvince: Khyber Pakhtunkhwa; locality: Abbottabad, Kaghan Colony; verbatimElevation: 1255 m; decimalLatitude: 34.19139; decimalLongitude: 73.22500; **Event:** year: 2015; month: 10; day: 21; **Record Level:** institutionID: PMAS-AAUR
- **Type status:** Other material. **Occurrence:** individualCount: 3; sex: 1 male, 2 females; occurrenceID: 9EEC3F37-D606-5781-8A87-1A63E2AE386E; **Taxon:** scientificName: *Coccinellatransversalis*; order: Coleoptera; family: Coccinellidae; scientificNameAuthorship: Fabricius, 1781; **Location:** country: Pakistan; stateProvince: Khyber Pakhtunkhwa; locality: Mansehra, Nisar Park; verbatimElevation: 1120 m; decimalLatitude: 34.34361; decimalLongitude: 73.21833; **Event:** year: 2015; month: 10; day: 22; **Record Level:** institutionID: PMAS-AAUR
- **Type status:** Other material. **Occurrence:** individualCount: 1; sex: 1 male; occurrenceID: 5BA68340-F7A0-5C8A-843B-F12DB735AED5; **Taxon:** scientificName: *Coccinellatransversalis*; order: Coleoptera; family: Coccinellidae; scientificNameAuthorship: Fabricius, 1781; **Location:** country: Pakistan; stateProvince: Punjab; locality: Chakwal, Dhudiyal; verbatimElevation: 507 m; decimalLatitude: 33.11000; decimalLongitude: 73.01249; **Event:** year: 2015; month: 12; day: 10; **Record Level:** institutionID: PMAS-AAUR
- **Type status:** Other material. **Occurrence:** individualCount: 3; sex: 1 male, 2 females; occurrenceID: 56C0B261-D824-5D4E-8318-AE846DF0B238; **Taxon:** scientificName: *Coccinellatransversalis*; order: Coleoptera; family: Coccinellidae; scientificNameAuthorship: Fabricius, 1781; **Location:** country: Pakistan; stateProvince: Khyber Pakhtunkhwa; locality: Abbottabad, Kaghan Colony; verbatimElevation: 1255 m; decimalLatitude: 34.19139; decimalLongitude: 73.22500; **Event:** year: 2018; month: 11; day: 1; **Record Level:** institutionID: PMAS-AAUR
- **Type status:** Other material. **Occurrence:** individualCount: 1; sex: 1 male; occurrenceID: 60B60BFB-EB8E-528E-8976-CB692DCC028D; **Taxon:** scientificName: *Coccinellatransversalis*; order: Coleoptera; family: Coccinellidae; scientificNameAuthorship: Fabricius, 1781; **Location:** country: Pakistan; stateProvince: Punjab; locality: Chakwal, Dhudiyal; verbatimElevation: 507 m; decimalLatitude: 33.11000; decimalLongitude: 73.01222; **Event:** year: 2021; month: 8; day: 10; **Record Level:** institutionID: PMAS-AAUR

#### Description

**Figure 4.**
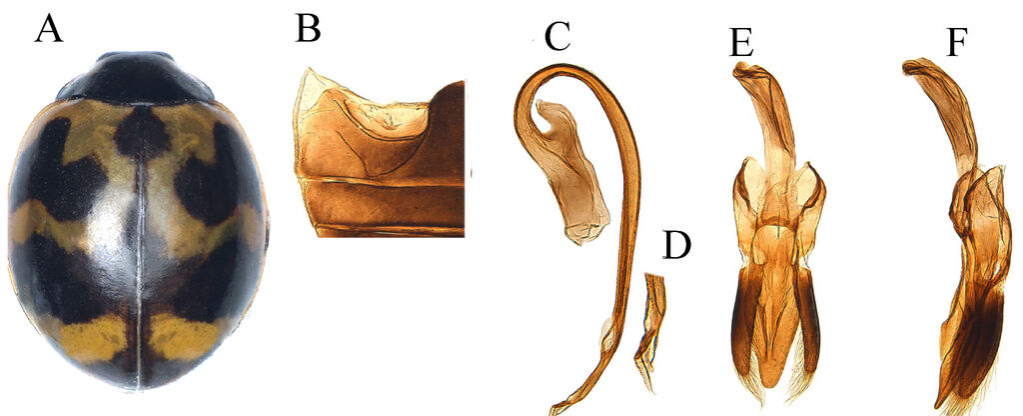
*Coccinellatransversalis* Fabricius: **A** dorsal view; **B** abdomen; **C** penis; **D** penis apex; **E** tegmen inner view; **F** tegmen lateral view.

Body (Fig. 4A) broadly oval and fairly convex, dorsum apparently glabrous; head black with triangular yellowish-white spots on each side of frons; mouthparts and antennae dark brown; pronotum black with white-yellow sub-quadrangular marking on anterior corners, connected at anterior margin; scutellar shield black; elytra orange-red, suture with narrow black band and sub-oval scutellar spot, three transverse markings on base, median and apical of each elytron, basal ones elbow-shape marks, but not touching base, median ones large and wave-like in shape, apical one transverse connected with sutural margin and sometimes entirely covering the apex of elytra as in Fig. 4A; underside and legs black; abdominal-postcoxal line (Fig. 4B) incomplete, not recurved and reaching to posterior margin ventrite 1, oblique dividing line present. Male genitalia as on Fig. 4C-F.

##### Measurements

TL: 5.30–547 mm; TW: 4.29–4.37 mm; HW: 1.35–1.39 mm; TH: 2.36–2.38 mm; EL/TW: 1.06–1.08; TL/TW: 1.24–1.26; PL/PW: 0.52–0.55.

#### Diagnosis

*Coccinellatransversalis* can easily be distinguished from *C.marussii* and *C.luteopicta* by having three transverse large markings on the elytra and apex of the penis with a slightly dilated and small thread-like appendage at the tip as in Fig. 4D.

#### Distribution

Pakistan. Azad Jammu and Kashmir (Khan et al. 1999, Rafi et al. 2005, Inayatullah et al. 2005, Khan et al. 2007, Hayat et al. 2017), Khyber Pakhtunkhwa (Ullah et al. 2012, Saeed et al. 2016, current study), Islamabad, Punjab (Rafi et al. 2005, current study), Sindh (Ali et al. 2012); – India, Bangladesh, Nepal, Japan, China, Indonesia and Sri Lanka, (Poorani 2002, Kovář 2007, Abdolahi et al. 2018).

#### Ecology

**Prey.** Rafi et al. (2005) reported that this species feeds on *Aphisgossypii* Glover, *A.fabae* Scopoli, *A.craccivora* Koch, *Myzuspersicae* (Sulzer), *Lipaphispseudobrassicae* Davis, *Macrosiphumrosae* (Linnaeus), *Pterochloroidespersicae* Cholodkovsky, *Therioaphistrifolii* (Monell) (Hemiptera, Aphididae). During this study, *Coccinellatransversalis* was found to feed on *Aphisfabae* Scopoli, *Aulacorthumsolani* (Kaltenbach) and *Sitobiongraminis* Takahashi (Hemiptera, Aphididae).

**Associated plants.** *Coccinellatransversalis* was collected from *Heteropogoncontortus* (Linnaeus) (Poales, Poaceae), *Tagetesminuta* Linnaeus (Asterales, Asteraceae) and *Cyperusrotundus* Linnaeus (Poales, Cyperaceae) (current study).

#### Notes

Kovář (2007) considered *C.transversalis* as a subspecies of *C.leonina Fabricius 1775*, a species solely endemic to New Zealand. This was followed by Abdolahi et al. (2018). However, Ślipiński et al. (2020) treated *C.leonina* and *C.transversalis* as distinct species in their revision of Australo-Pacific Coccinellini.

### Coccinella iranica

Dobzhansky, 1926

7A43994D-2088-5838-97B2-FD2A9E29B146

Coccinella iranica Dobzhansky, 1926: 26; Mader (1930): 165; Korschefsky (1932): 467; Ashfaque et al. (2013): 241.
Coccinella (s. str.) iranica : Iablokoff-Khnzorian (1979): 68, Iablokoff-Khnzorian (1982): 382.

#### Materials

- **Type status:** Other material. **Occurrence:** individualCount: 1; sex: 1 female; occurrenceID: 3E97138F-A6D4-5E95-A1B5-E8232D985CA5; **Taxon:** scientificName: *Coccinellairanica*; order: Coleoptera; family: Coccinellidae; scientificNameAuthorship: Dobzhansky, 1926; **Location:** country: Pakistan; stateProvince: Gilgit-Baltistan,; locality: Hunza, Duikar; verbatimElevation: 2821 m; decimalLatitude: 36.32505; decimalLongitude: 74.6903; **Identification:** identificationReferences: Ashfaque et al. 2013; **Event:** year: 2005; month: 4; day: 20; **Record Level:** institutionID: NIM

#### Description

Body broadly oval and slightly convex, dorsal surface glabrous; head black; mouthparts and antennae dark brown to black; pronotum mostly black with a brownish stripe along anterior margins; scutellar shield black; elytra brown to brownish-yellow with eight irregular black spots, first elongate oval black spot at anterior margin along suture of both elytrons, second large L-shaped black mark at the middle, third oval spot at posterolateral margins and fourth sub-equal black spot near suture at the posterior margin of elytra. Male genitalia: penis apex flattened, with blunt tip; penis guide in inner view with narrow and rounded tip, paramers slightly shorter than the penis guide (Ashfaque et al. 2013).

##### Measurements

TL: 5.30–547 mm; TW: 4.29–4.37 mm (Ashfaque et al. 2013).

#### Diagnosis

*Coccinellairanica* is similar to *C.marussii*, but it can be differentiated by the latter from the brownish stripes on the anterior margins and the penis guide with the narrow and rounded tip in the inner view. In *C.marussii*, pronotal anterior corners with pale yellow quadrangular marks, penis guide with a broad and blunt tip in the inner view (Fig. 3E).

#### Distribution

Pakistan: Gilgit-Baltistan (Ashfaque et al. 2013); – Iran (Dobzhansky 1926, Kovář 2005), Turkey (Fürsch 1981, Kovář 2005).

#### Ecology

**Prey.** Unknown from Pakistan.

**Associated plants.** Unknown from Pakistan.

#### Notes

Dobzhansky (1926) described and illustrated this species for the first time from northern Iran. Later on, Dobzhansky (1931) considered it under the *Coccinelladifficilis* species group. Fürsch (1981) described and illustrated its male genitalia. Ashfaque et al. (2013) reported this species from Pakistan.

### Coccinella septempunctata

Linnaeus, 1758

558E7938-FF16-5C0A-913C-C87AD0BCB999

Coccinella septempunctata Linnaeus, 1758: 365: *Korschefsky (1932)*: 486; Gordon and Vandenberg (1991): 847; Yu and Wang (1999): 94; Yu et al. (2000): 193; Irshad (2001): 1259; Poorani (2002): 21; Rafi et al. (2005): 45;: 188; Yu (2011): 45; Ali et al. (2012): 168; Ashfaque et al. (2013): 243.
Coccinella divaricata Olivier, 1808: 1001; Korschefsky (1932): 457; Mader (1930): 375; Gordon (1987): 13.
Coccinella confusa Wiedemann, 1823: 72; Mulsant (1850): 112 (as a var. of *divaricata*).
Coccinella brucki Mulsant, 1866: 90; Crotch (1874): 46.

#### Materials

- **Type status:** Other material. **Occurrence:** individualCount: 10; sex: 3 males, 7 females; occurrenceID: 61EA3796-071E-5D44-AA56-F70D67AC9873; **Taxon:** scientificName: *Coccinellaseptempunctata*; order: Coleoptera; family: Coccinellidae; scientificNameAuthorship: Linnaeus 1758; **Location:** country: Pakistan; stateProvince: Punjab; locality: Attock, Hazro; verbatimElevation: 317 m; decimalLatitude: 33.89500; decimalLongitude: 72.48361; **Event:** samplingProtocol: mist net; year: 2014; month: 10; day: 1; **Record Level:** institutionID: PMAS-AAUR
- **Type status:** Other material. **Occurrence:** individualCount: 3; sex: 1 male, 2 females; occurrenceID: F4F34E53-0E0E-5520-A98E-1A7F27F42E49; **Taxon:** scientificName: *Coccinellaseptempunctata*; order: Coleoptera; family: Coccinellidae; scientificNameAuthorship: Linnaeus 1758; **Location:** country: Pakistan; stateProvince: Punjab; locality: Rawalpindi, Neela Sandh; verbatimElevation: 692 m; decimalLatitude: 33.66056; decimalLongitude: 73.38417; **Event:** samplingProtocol: mist net; year: 2015; month: 4; day: 9; **Record Level:** institutionID: PMAS-AAUR
- **Type status:** Other material. **Occurrence:** individualCount: 2; sex: 1 male, 1 female; occurrenceID: 19F2E70A-D2C5-5A9F-A9FB-1172F7006397; **Taxon:** scientificName: *Coccinellaseptempunctata*; order: Coleoptera; family: Coccinellidae; scientificNameAuthorship: Linnaeus 1758; **Location:** country: Pakistan; stateProvince: Punjab; locality: Jhelum, Dina; verbatimElevation: 277 m; decimalLatitude: 33.03000; decimalLongitude: 73.59277; **Event:** samplingProtocol: mist net; year: 2015; month: 4; day: 21; **Record Level:** institutionID: PMAS-AAUR
- **Type status:** Other material. **Occurrence:** individualCount: 6; sex: 3 males, 3 females; occurrenceID: BF968727-2103-5057-88C2-CEA282CB0E84; **Taxon:** scientificName: *Coccinellaseptempunctata*; order: Coleoptera; family: Coccinellidae; scientificNameAuthorship: Linnaeus 1758; **Location:** country: Pakistan; stateProvince: Punjab; locality: Chakwal, Dhudiyal; verbatimElevation: 507 m; decimalLatitude: 33.11000; decimalLongitude: 73.01222; **Event:** samplingProtocol: mist net; year: 2015; month: 5; day: 14; **Record Level:** institutionID: PMAS-AAUR
- **Type status:** Other material. **Occurrence:** individualCount: 21; sex: 8 males, 13 females; occurrenceID: 80382456-22AF-513D-B27E-2228494A9776; **Taxon:** scientificName: *Coccinellaseptempunctata*; order: Coleoptera; family: Coccinellidae; scientificNameAuthorship: Linnaeus 1758; **Location:** country: Pakistan; stateProvince: Punjab; locality: Murree, Kuldana; verbatimElevation: 1929 m; decimalLatitude: 33.92278; decimalLongitude: 73.40389; **Event:** samplingProtocol: mist net; year: 2016; month: 11; day: 11; **Record Level:** institutionID: PMAS-AAUR
- **Type status:** Other material. **Occurrence:** individualCount: 5; sex: 1 male, 4 females; occurrenceID: 878644C8-0A79-517E-8FF7-6AEBC5DE2E68; **Taxon:** scientificName: *Coccinellaseptempunctata*; order: Coleoptera; family: Coccinellidae; scientificNameAuthorship: Linnaeus 1758; **Location:** country: Pakistan; stateProvince: Islamabad; locality: Ankara Park; verbatimElevation: 504 m; decimalLatitude: 33.69139; decimalLongitude: 73.11194; **Event:** samplingProtocol: mist net; year: 2017; month: 11; day: 28; **Record Level:** institutionID: PMAS-AAUR
- **Type status:** Other material. **Occurrence:** individualCount: 9; sex: 4 males, 5 females; occurrenceID: 288B917F-E5A6-5D45-87DD-C2953285E817; **Taxon:** scientificName: *Coccinellaseptempunctata*; order: Coleoptera; family: Coccinellidae; scientificNameAuthorship: Linnaeus 1758; **Location:** country: Pakistan; stateProvince: Gilgit-Baltistan; locality: Gilgit, Jutial Nala; verbatimElevation: 1815 m; decimalLatitude: 35.88889; decimalLongitude: 74.34361; **Event:** samplingProtocol: mist net; year: 2018; month: 8; day: 29; **Record Level:** institutionID: PMAS-AAUR
- **Type status:** Other material. **Occurrence:** individualCount: 8; sex: 4 males, 4 females; occurrenceID: 1EE70DDA-4329-53F4-A721-7A88F4E44301; **Taxon:** scientificName: *Coccinellaseptempunctata*; order: Coleoptera; family: Coccinellidae; scientificNameAuthorship: Linnaeus 1758; **Location:** country: Pakistan; stateProvince: Azad Jammu and Kashmir; locality: Muzaffarabad; verbatimElevation: 682 m; decimalLatitude: 34.36056; decimalLongitude: 73.47639; **Event:** samplingProtocol: mist net; year: 2018; month: 6; day: 21; **Record Level:** institutionID: PMAS-AAUR
- **Type status:** Other material. **Occurrence:** individualCount: 21; sex: 8 males, 13 females; occurrenceID: 454BB03E-2C9A-5C40-9717-9083B1707FA4; **Taxon:** scientificName: *Coccinellaseptempunctata*; order: Coleoptera; family: Coccinellidae; scientificNameAuthorship: Linnaeus 1758; **Location:** country: Pakistan; stateProvince: Khyber Pakhtunkhwa; locality: Abbottabad, Khanaspur; verbatimElevation: 2362 m; decimalLatitude: 34.02056; decimalLongitude: 73.42583; **Event:** samplingProtocol: mist net; year: 2017; month: 5; day: 4; **Record Level:** institutionID: PMAS-AAUR
- **Type status:** Other material. **Occurrence:** individualCount: 4; sex: 1 male, 5 females; occurrenceID: A47AEE9C-6D78-5D00-BE43-CAC1E51FEF87; **Taxon:** scientificName: *Coccinellaseptempunctata*; order: Coleoptera; family: Coccinellidae; scientificNameAuthorship: Linnaeus 1758; **Location:** country: Pakistan; stateProvince: Azad Jammu and Kashmir; locality: Neelum, Sharda; verbatimElevation: 1967 m; decimalLatitude: 34.79389; decimalLongitude: 74.18167; **Event:** samplingProtocol: mist net; year: 2019; month: 8; day: 20; **Record Level:** institutionID: PMAS-AAUR
- **Type status:** Other material. **Occurrence:** individualCount: 7; sex: 3 males, 4 females; occurrenceID: BB4D077F-FE96-5521-969F-7C794E3D955D; **Taxon:** scientificName: *Coccinellaseptempunctata*; order: Coleoptera; family: Coccinellidae; scientificNameAuthorship: Linnaeus 1758; **Location:** country: Pakistan; stateProvince: Gilgit-Baltistan; locality: Hunza, Aliabad; verbatimElevation: 2222 m; decimalLatitude: 36.30611; decimalLongitude: 74.61833; **Event:** samplingProtocol: mist net; year: 2020; month: 7; day: 5; **Record Level:** institutionID: PMAS-AAUR
- **Type status:** Other material. **Occurrence:** individualCount: 1; sex: 1 male; occurrenceID: 0CA59878-8B92-553C-B1D9-13C0BFCD26A4; **Taxon:** scientificName: *Coccinellaseptempunctata*; order: Coleoptera; family: Coccinellidae; scientificNameAuthorship: Linnaeus 1758; **Location:** country: Pakistan; stateProvince: Gilgit-Baltistan; locality: Skardu, Kachura; verbatimElevation: 2342 m; decimalLatitude: 35.43472; decimalLongitude: 75.45000; **Event:** samplingProtocol: mist net; year: 2021; month: 4; day: 5; **Record Level:** institutionID: PMAS-AAUR
- **Type status:** Other material. **Occurrence:** individualCount: 6; sex: 3 males, 3 female; occurrenceID: 1B73ED17-38E8-509B-830D-AD6E0FDD7E63; **Taxon:** scientificName: *Coccinellaseptempunctata*; order: Coleoptera; family: Coccinellidae; scientificNameAuthorship: Linnaeus 1758; **Location:** country: Pakistan; stateProvince: Gilgit-Baltistan; locality: Astore, Rama; verbatimElevation: 3093 m; decimalLatitude: 35.35889; decimalLongitude: 74.81111; **Event:** samplingProtocol: mist net; year: 2022; month: 8; day: 22; **Record Level:** institutionID: PMAS-AAUR

#### Description

**Figure 5.**
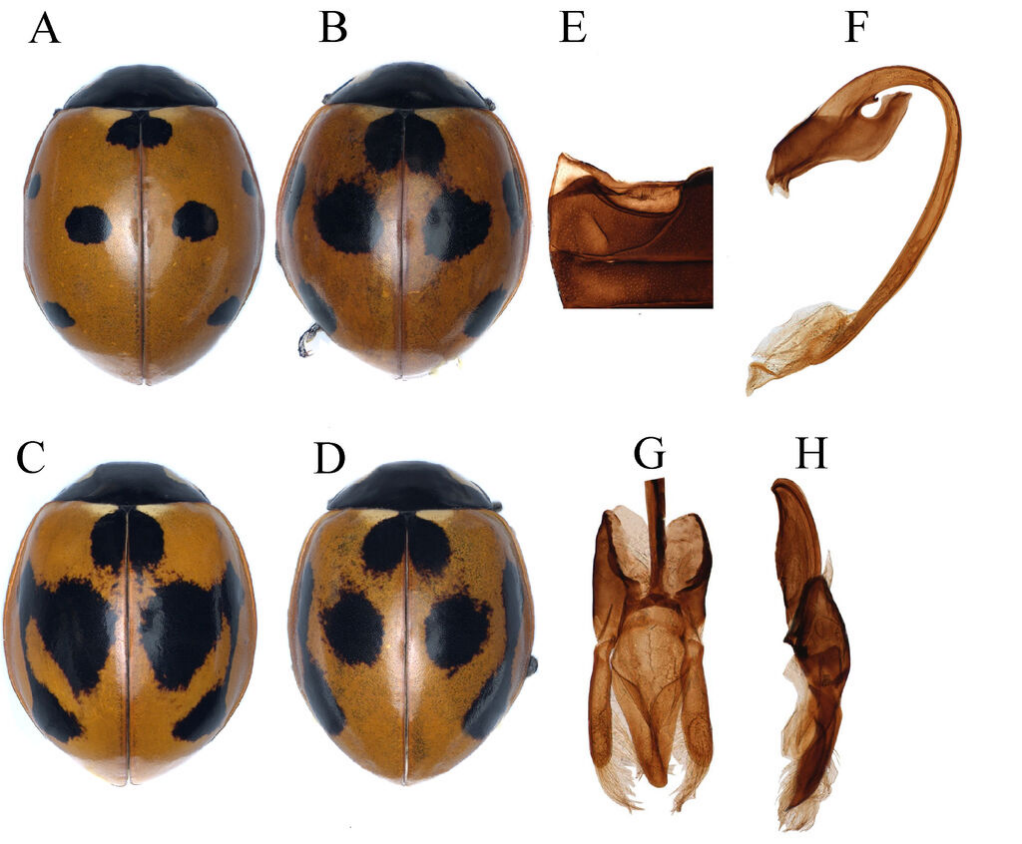
*Coccinellaseptempunctata* Linnaeus. **A-D** dorsal view; **E** abdomen; **F** penis; **G** tegmen inner view; **H** tegmen lateral view.

Body (Fig. 5A-D) oval, strongly convex and dorsum surface apparently glabrous; head black with pale yellow oblique spots near ocular margins, antennae and mouth-parts black; scutellum black; pronotum black with pale-yellow quadrated marking at anterior half of lateral margins; elytra reddish-brown or orange, with seven black spots, each spot nearly rounded and rather large, sometimes marging with each other and forming one or two large black spots, in different patterns; abdominal postcoxal line (Fig. 5E) incomplete laterally and not recurved, expending to posterior margin ventrite 1, with an oblique dividing line close to postcoxal line. Male genitalia as in Fig. 5F-H.

##### Measurements

TL: 6.63–7.20 mm; TW: 5.35–5.60 mm; HW: 0.86–0.94 mm; TH: 3.26–3.28 mm; EL/TW: 1.07–1.09; TL/TW: 1.24–1.33; PL/PW: 0.42–0.44; PW/EW: 0.63–0.65.

#### Diagnosis

*Coccinellaseptempunctata* can be easily separated from *C.transversoguttata* and *C.undecimpunctata* by having seven black spots at the elytra (Fig. 5A-B), sometimes these spots merging and forming one or two large black marks at each elytron (Fig. 5C-D); apex of penis flattened and with the triangular process, surrounded by a weak membrane (Fig. 5F).

#### Distribution

It is a widely distributed species in Pakistan (Rafi et al. 2005). Azad Jammu & Kashmir (Hayat et al. 2017, current study); Baluchistan (Mohibullah et al. 2019), Gilgit-Baltistan (Ashfaque et al. 2013, current study), Punjab (Khan and Sultan 1949, Gillani 1976, Ashfaque et al. 2013, Ahmed et al. 2017, current Study), Islamabad (current study), Khyber Pakhtunkhwa (Mohyudddin 1981, Shah 1983, Ullah et al. 2012, Saeed et al. 2016, Khan et al. 2007, current study), Sindh (Ali et al. 2012, Ashfaque et al. 2013); – China, Nepal, Bhutan, India, Iran, Sri Lanka, Andorra, Austria, Azores, Balearic, Albania, Liechtenstein, Belarus, Cyprus, Netherlands, Belgium, Romania, Estonia, Bosnia, Herzegovina, Finland, Germany, Afro-tropical Region, Bulgaria, Corsica, Croatia, England, Sweden, France, Greek, Hungary, Italy, Lithuania, Denmark, Macedonia, Madeira, Czech Republic, Malta, Norway, Slovenia, Ireland, Poland, Luxembourg, Ukraine, Portuguese, Russia, Sardinia, Latvia, Slovakia, Spain, Switzerland and East Palearctic, all being introduced to the Nearctic Region (Poorani 2002, Kovář 2007, Jafari et al. 2015).

#### Ecology

**Prey.** Irshad (2001) and Rafi et al. (2005) recorded that this species feeding on *Aleurocanthushusaini* (Corbett), *A.woglumi* Ashby, *Aleurolobusbarodensis* (Maskell), *Bemisiatabaci* (Gennadius), *Dialeurodescitri* (Ashmead), *Neomaskellia* spp. (Hemiptera, Aleyrodidae), *Adelgesjoshii* (Schneider-Orelli and Schneider) (Hemiptera, Adelgididae), *Acyrthosiphonpisum* Harris, *Therioaphistrifolii* (Monell), *Aphisgossypii* Glover, *A.fabae* Scopoli, *A.craccivora* Koch, *Capitophorus* sp., *Myzuspersicae* (Sulzer), *Lipaphispseudobrassicae* Davis, *Pterochloroidespersicae* Cholodkovsky, *Macrosiphumrosae* (Linnaeus), *Rhopalosiphummaidis* (Fitch) (Hemiptera, Aphididae), *Diaphorinacitri* Kuwayama (Hemiptera, Psyllidae), *Pyrillapurposilla* (Walker) (Hemiptera, Lophopidae), *Centrococcusinsolitus* Ferris, *Quadraspidiotusperniciosus* Comstock (Hemiptera, Diaspididae) and *Coccusviridis* (Green) (Hemiptera, Coccidae), *Coccidohystrixinsolita* (Green) and *Ferrisiavirgata* (Cockerell) (Hemiptera, Pseudococcidae) (Irshad 2001, Rafi et al. 2005).

In this study, this species was found feeding on *Acyrthosiphonloti* (Theobald), *Aulacorthumsolani* (Kaltenbach), *A.pisum* Harris, *Aphiscraccivora* Koch, *A.fabae* Scopoli, *A.farinose* (Gmelin), *A.gossypii* Glover, *A.spiraecola*, *A.solanella* Theobald, *A.punicae* Passerini, *A.verbasci* Schrank, *Brachycaudushelichrysi* (Kaltenbach), *Brevicorynebrassicae* (Linnaeus), *Capitophorusformosartemisae* (Takahashi), *Chaitophoruspopulifolii* Essig, *Cinaratujafilina* (Del Guercio), *Eriosomalanigerum* (Hausmann), *Hysteroneurasetariae* (Thomas), *Lachnuswichmanni* Hille Ris Lambers, *Lipaphiserysimi* (Kaltenbach), *Macrosiphumpachysiphon* Hille Ris Lambers, *M.rosae* (Linnaeus), *Meldnaphisdonacis* (Passerini), *Myzocallispakistanicus* Hille Ris Lambers, *Myzusornatus* (Laing), *Myzuspersicae* (Sulzer), *Periphyllus* sp., *Phorodoncannabis* Passerini, *Rhopalosiphumpadi* (Linnaeus), *R.maidis* (Fitch), *Shivaphiscelti* Das, *Schizaphisgraminum* (Rondani), *Sitobionavenae* Fabricius, *Tinocalliskahawaluokalani* (Kirkaldy), *Therioaphistrifolii* (Monell), *Toxopteraaurantii* (Boyer de Fonscolombe), *Tuberolachnussalignus* Gmelin, *Uroleuconcarthami* (Theobald) (Hemiptera, Aphididae), Adelges (Dreyfusia) knucheli (Schneider-Orelli and Schneider) (Hemiptera, Adelgidae), *Drosichastebbingi* Green), *D.mangiferae* (Green) (Hemiptera, Margarodidae), *Planococcuscitri* (Risso), *Phenacoccussolenopsis* Tinsely, *Maconellicoccushirsutus* (Green) (Hemiptera, Pseudococcidae), *Dialeurodescitri* (Ashmead) (Hemiptera, Aleyrodidae) *Camarotoscena* sp., *Heteropsylla* sp. (Hemiptera, Psyllidae), some species of scale insects (Hemiptera, Diaspididae) and *Phyllocnistis* sp. (Lepidoptera, Gracillariidae).

**Associated Plants.** This species was collected from *Cumiumcyminum* (Linnaeus) (Apiales, Apiaceae), *Anthemisarvensis* (Linnaeus), *A.tinctoria* Kelwayi, *Artemisia* sp., *A.maritima* (Linnaeus), *Carthamusoxyacantha* Bieberstein, *Erigeroncanadensis* (Linnaeus), *Partheniumhysterophorus* (Linnaeus), *Sonchusasper* (Linnaeus) Hill, *Tagetesminuta* (Linnaeus) (Asterales, Asteraceae), *Brassicacampestris* (Linnaeus), B.campestrisvarbotrytis (Linnaeus), *B.oleracea* (Linnaeus) (Brassicales, Brassicaceae), *Capparisspinosa* Linnaeus (Brassicales, Capparaceae), *Moringaoleifera* Lamarck (Brassicales, Moringaceae), *Spinaciaoleracea* (Linnaeus) (Caryophyllales, Amaranthaceae), *Bougainvilleaglabra* Choisy, *Mirabilisjalapa* (Linnaeus) (Caryophyllales, Nyctaginaceae), *Abiespindrow* (Royle ex Don) (Pinales, Pinaceae), *Rumexdentatus* (Linnaeus), *R.hastatus* Don (Caryophyllales, Polygonaceae), *Tamarixgallica* (Linnaeus) (Caryophyllales, Tamaricaceae), *Quercusbaloot* Griffith (Fagales, Fagaceae), *Acaciafarnesiana* Wight et Arnott, *Cassiafistula* Linnaeus, *Dalbergiasissoo* Roxburgh, *Medicagosativa* Linnaeus, *Prosopisjuliflora* (Swartz) de-Candolle, *Sophoraalopecuroides* Linnaeus, *Trifoliumalexandrinum* Linnaeus (Fabales, Fabaceae), *Juglansregia* Linnaeus (Fagales, Juglandaceae), *Clerodendrumphilippinum* (Osbeck) Mabberley (Lamiales, Lamiaceae), *Verbascumthapsus* Linnaeus (Lamiales, Scrophulariaceae), *Oleaeuropaea* Linnaeus (Lamiales, Oleaceae), *Durantaerecta* Linnaeus, *Lantanacamara* Linnaeus (Lamiales, Verbenaceae), *Hibiscusrosa-sinensis* Linnaeus (Malvales, Malvaceae), *Populusnigra* Linnaeus, *Salixalba* Linnaeus (Malpighiales, Salicaceae), *Lagerstroemiaindica* Linnaeus, *Punicagranatum* Linnaeus (Myrtales, Lythraceae), *Thuja* sp. (Pinales, Cupressaceae), *Psidiumguajava* Linnaeus, *Syzygiumcumini* (Linnaeus) Skeels (Myrtales, Myrtaceae), *Cyperusrotundus* Linnaeus (Poales, Cyperaceae), *Avenasativa* Linnaeus, *Arundodonax* Linnaeus, *Cynodondactylon* (Linnaeus) Persoon, *Dactylocteniumaegyptium* (Linnaeus) Willdenow, *Heteropogoncontortus* (Linnaeus) Beauvois ex Roemer and Schultes, *Phragmiteskarka* (Retzius), *Triticumaestivum* Linnaeus, (Poales, Poaceae), *Berberislyceum* Royle (Ranunculales, Berberidaceae), *Cannabissativa* Linnaeus, *Celtiseriocarpa* Decne. (Rosales, Cannabaceae), *Eriobotryajaponica* (Thunberg) Lindley, *Hippophaerhamnoides* Linnaeus (Rosales, Elaeagnaceae), *Malusdomestica* Borkhausen, *Prunusavium* Linnaeus, *P.armeniaca* Linnaeus, *P.persica* (Linnaeus) Batsch, *Pyruscommunis* Linnaeus, *P.pyrifolia* (Burman) Nakai, *Rosaindica* Trattinnick (Rosales, Rosaceae), *Ficus* sp., *Morusalba* Linnaeus (Rosales, Moraceae), *Aesculusindica* (Wallich ex Cambessèdes) Hooker (Sapindales, Sapindaceae), *Citrus* sp. (Sapindales, Rutaceae), *Convolvulusarvensis* Linnaeus (Solanales, Convolvulaceae), *Cestrumdiurnum* Linnaeus, *Solanumnigrum* Linnaeus, *S.tuberosum* Linnaeus, *Withaniasomnifera* (Linnaeus) Dunal (Solanales, Solanaceae), *Ziziphusmauritiana* Lamarck, *Z.nummularia* (Burman) Wight and Arnott (Rosales, Rhamnaceae) and *Urticadioica* Linnaeus (Rosales, Urticaceae) (current study).

### Coccinella transversoguttata transversoguttata

Faldermann, 1835

4A00A233-3474-5BE5-9B17-C2F3AEF6A57C

Coccinella transversoguttata Faldermann, 1835: 454: Mulsant (1850): 117; Crotch (1874): 116; Weise (1885): 28; Jacobson (1915): 982; Dobzhansky (1926): 21; Mader (1930): 150; Iablokoff-Khnzorian (1979): 67; Fürsch (1981): 82; Iablokoff-Khnzorian (1982): 364; Bielawski (1984): 417; Poorani (2002): 327; Kovář (2005): 149; Ren et al. (2009): 188; Ashfaque et al. (2013): 245; Poorani (2023): 91.
Coccinella transversoguttata var. *Sedakovi* Weise, 1889: 537.
Coccinella geminopunctata Liu, 1962: 265: Iablokoff-Khnzorian (1979): 68; Iablokoff-Khnzorian (1982): 362.

#### Materials

- **Type status:** Other material. **Occurrence:** individualCount: 5; sex: 2 males, 3 females; occurrenceID: 050C38E8-D35B-55DF-82A6-57329E904A42; **Taxon:** scientificName: Coccinellatransversoguttatatransversoguttata; order: Coleoptera; family: Coccinellidae; scientificNameAuthorship: Faldermann, 1835; **Location:** country: Pakistan; stateProvince: Gilgit-Baltistan; locality: Ghanche, Barah; verbatimElevation: 2466 m; decimalLatitude: 35.20556; decimalLongitude: 76.27444; **Event:** samplingProtocol: mist net; year: 2015; month: 7; day: 13; **Record Level:** institutionID: PMAR-AAU
- **Type status:** Other material. **Occurrence:** individualCount: 1; sex: 1 male; occurrenceID: 7646AEA8-A4D7-5142-A3CD-4F6B1989FFB5; **Taxon:** scientificName: Coccinellatransversoguttatatransversoguttata; order: Coleoptera; family: Coccinellidae; scientificNameAuthorship: Faldermann, 1835; **Location:** country: Pakistan; stateProvince: Gilgit-Baltistan; locality: Ghanche, Khapulo; verbatimElevation: 2671 m; decimalLatitude: 35.15222; decimalLongitude: 76.335; **Event:** samplingProtocol: mist net; year: 2015; month: 7; day: 13; **Record Level:** institutionID: PMAR-AAU
- **Type status:** Other material. **Occurrence:** individualCount: 1; sex: 1 male; occurrenceID: DBA275C1-CA5C-5316-965B-DD749154F96B; **Taxon:** scientificName: Coccinellatransversoguttatatransversoguttata; order: Coleoptera; family: Coccinelidae; scientificNameAuthorship: Faldermann, 1835; **Location:** country: Pakistan; stateProvince: Gilgit-Baltistan; locality: Ghanche, Keris; verbatimElevation: 2284 m; decimalLatitude: 35.23361; decimalLongitude: 75.94028; **Event:** samplingProtocol: mist net; year: 2015; month: 7; day: 14; **Record Level:** institutionID: PMAR-AAU
- **Type status:** Other material. **Occurrence:** individualCount: 2; sex: 1 male, 1 female; occurrenceID: AA8990E9-8FED-52AD-A30B-9AFBAB5E6251; **Taxon:** scientificName: Coccinellatransversoguttatatransversoguttata; order: Coleoptera; family: Coccinellidae; scientificNameAuthorship: Faldermann, 1835; **Location:** country: Pakistan; stateProvince: Gilgit-Baltistan; locality: Skardu, Gol; verbatimElevation: 2337 m; decimalLatitude: 35.25083; decimalLongitude: 75.865; **Event:** samplingProtocol: mist net; year: 2015; month: 8; day: 5; **Record Level:** institutionID: PMAR-AAU
- **Type status:** Other material. **Occurrence:** individualCount: 2; sex: 1male, 1 female; occurrenceID: 29EE074B-6E9D-53D2-B165-4DB5F3DE9785; **Taxon:** scientificName: Coccinellatransversoguttatatransversoguttata; order: Coleoptera; family: Coccinellidae; scientificNameAuthorship: Faldermann, 1835; **Location:** country: Pakistan; stateProvince: Gilgit-Baltistan; locality: Ghizer, Iskoman, Shones; verbatimElevation: 2236 m; decimalLatitude: 36.47333; decimalLongitude: 73.84722; **Event:** samplingProtocol: mist net; year: 2015; month: 7; day: 22; **Record Level:** institutionID: PMAR-AAU
- **Type status:** Other material. **Occurrence:** individualCount: 1; sex: 1 female; occurrenceID: F648B22E-FA46-530A-A0FF-136BBDE3F5F6; **Taxon:** scientificName: Coccinellatransversoguttatatransversoguttata; order: Coleoptera; family: Coccinellidae; scientificNameAuthorship: Faldermann, 1835; **Location:** country: Pakistan; stateProvince: Gilgit-Baltistan; locality: Ghizer, Gupis, Shamaran; verbatimElevation: 2625 m; decimalLatitude: 36.17722; decimalLongitude: 72.98555; **Event:** samplingProtocol: mist net; year: 2015; month: 8; day: 4; **Record Level:** institutionID: PMAR-AAU
- **Type status:** Other material. **Occurrence:** individualCount: 7; sex: 3 males, 4 females; occurrenceID: ED168A38-6626-531E-8CB0-E3C2BA1B0849; **Taxon:** scientificName: Coccinellatransversoguttatatransversoguttata; order: Coleoptera; family: Coccinellidae; scientificNameAuthorship: Faldermann, 1835; **Location:** country: Pakistan; stateProvince: Gilgit-Baltistan; locality: Ghizer, Yasin, Hunder; verbatimElevation: 2500 m; decimalLatitude: 36.51376; decimalLongitude: 73.41472; **Event:** samplingProtocol: mist net; year: 2015; month: 8; day: 5; **Record Level:** institutionID: PMAR-AAU
- **Type status:** Other material. **Occurrence:** individualCount: 3; sex: 1 male, 2 females; occurrenceID: 652BAA3D-E959-5E00-BB04-BAE6D85E44D6; **Taxon:** scientificName: Coccinellatransversoguttatatransversoguttata; order: Coleoptera; family: Coccinellidae; scientificNameAuthorship: Faldermann, 1835; **Location:** country: Pakistan; stateProvince: Khyber Pakhtunkhwa; locality: Chitral, Booni; verbatimElevation: 2621 m; decimalLatitude: 36.24917; decimalLongitude: 72.25944; **Event:** samplingProtocol: mist net; year: 2017; month: 7; day: 20; **Record Level:** institutionID: PMAR-AAU
- **Type status:** Other material. **Occurrence:** individualCount: 1; sex: 1 male; occurrenceID: 721E9E31-8733-5ECD-B3B5-49A0343566E4; **Taxon:** scientificName: Coccinellatransversoguttatatransversoguttata; order: Coleoptera; family: Coccinellidae; scientificNameAuthorship: Faldermann, 1835; **Location:** country: Pakistan; stateProvince: Gilgit-Baltistan; locality: Ghanche, Ghorg; verbatimElevation: 2439 m; decimalLatitude: 35.31583; decimalLongitude: 75.86417; **Event:** samplingProtocol: mist net; year: 2017; month: 8; day: 9; **Record Level:** institutionID: PMAR-AAU
- **Type status:** Other material. **Occurrence:** individualCount: 1; sex: 1 male; occurrenceID: 1A6AB1B5-172C-51AB-9180-3960685BFD45; **Taxon:** scientificName: Coccinellatransversoguttatatransversoguttata; order: Coleoptera; family: Coccinellidae; scientificNameAuthorship: Faldermann, 1835; **Location:** country: Pakistan; stateProvince: Gilgit-Baltistan; locality: Ghanche, Keris; verbatimElevation: 2284 m; decimalLatitude: 35.23361; decimalLongitude: 75.94028; **Event:** samplingProtocol: mist net; year: 2015; month: 7; day: 14; **Record Level:** institutionID: PMAR-AAU
- **Type status:** Other material. **Occurrence:** individualCount: 3; sex: 1 male, 2 females; occurrenceID: 0FE34CBF-0EFD-5FBE-87E4-A8440449BA38; **Taxon:** scientificName: Coccinellatransversoguttatatransversoguttata; order: Coleoptera; family: Coccinellidae; scientificNameAuthorship: Faldermann, 1835; **Location:** country: Pakistan; stateProvince: Gilgit-Baltistan; locality: Hunza, Aliabad; verbatimElevation: 2222 m; decimalLatitude: 36.30611; decimalLongitude: 74.61833; **Event:** samplingProtocol: mist net; year: 2018; month: 6; day: 2; **Record Level:** institutionID: PMAR-AAU
- **Type status:** Other material. **Occurrence:** individualCount: 13; sex: 3 males, 10 females; occurrenceID: 0BBB6FDA-08A5-55D9-8BFD-DDA7AC973DD4; **Taxon:** scientificName: Coccinellatransversoguttatatransversoguttata; order: Coleoptera; family: Coccinellidae; scientificNameAuthorship: Faldermann, 1835; **Location:** country: Pakistan; stateProvince: Gilgit-Baltistan; locality: Ghizer, Punyial, Gahkuch-Bala; verbatimElevation: 2129 m; decimalLatitude: 37.51; decimalLongitude: 73.78; **Event:** samplingProtocol: mist net; year: 2019; month: 8; day: 3; **Record Level:** institutionID: PMAR-AAU
- **Type status:** Other material. **Occurrence:** individualCount: 16; sex: 9 males, 7 females; occurrenceID: C0700BF0-E031-56C3-A6F9-B84E5D7FAC50; **Taxon:** scientificName: Coccinellatransversoguttatatransversoguttata; order: Coleoptera; family: Coccinellidae; scientificNameAuthorship: Faldermann, 1835; **Location:** country: Pakistan; stateProvince: Gilgit-Baltistan; locality: Skardu, Hussainabad; verbatimElevation: 2313 m; decimalLatitude: 35.18028; decimalLongitude: 75.90694; **Event:** samplingProtocol: mist net; year: 2022; month: 5; day: 22; **Record Level:** institutionID: PMAR-AAU
- **Type status:** Other material. **Occurrence:** individualCount: 2; sex: 2 males; occurrenceID: 8444405C-28F7-5617-90BD-0EBA50C1994A; **Taxon:** scientificName: Coccinellatransversoguttatatransversoguttata; order: Coleoptera; family: Coccinellidae; scientificNameAuthorship: Faldermann, 1835; **Location:** country: Pakistan; stateProvince: Gilgit-Baltistan; locality: Skardu, Olding; verbatimElevation: 2253m; decimalLatitude: 35.2925; decimalLongitude: 75.6538; **Event:** samplingProtocol: mist net; year: 2022; month: 6; day: 19; **Record Level:** institutionID: PMAR-AAU

#### Description

**Figure 6.**
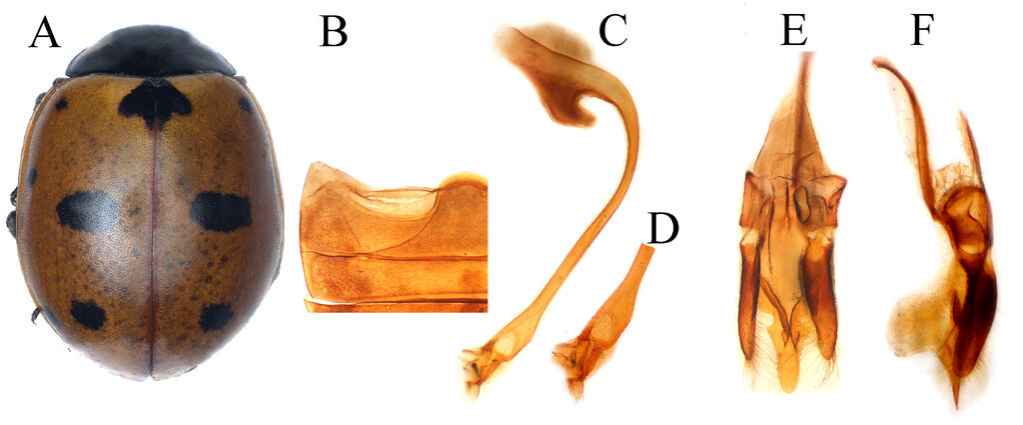
*Coccinellatransversoguttatatransversoguttata* Faldermanna: **A** dorsal view; **B** abdomen; **C** penis; **D** penis apex; **E** tegmen inner view; **F** tegmen lateral-view.

Body (Fig. 6A) broadly oval and dorsum glabrous; head black with large roundly triangular white-yellow to pale-yellow spots on each side of frons, connected with the inner orbit of eyes; mouthparts with antennae black; scutellum black; pronotum black with anterior corners white-yellow, connected at anterior margins; elytra reddish-orange, with eleven black spots, scutellar spot triangular, emarginate posteriorly along the suture, humeral spot small, lateral spots small, sub-rounded and slightly closer to lateral margin, discal spot large and transversely oval to sub-quadrate, marginal spots small and sub-rounded, apical spot moderately large and rounded; underside black, apical of propleura and mesepimera white at anterolateral corners, metepimera dark brown with whitish stripe; Male genitalia can be seen in Fig. 6C-F.

##### Measurements

TL: 5.23–6.43 mm; TW: 3.95–5.00 mm; HW: 1.28–1.52 mm; TH: 2.15–2.40 mm; EL/TW: 1.10–1.14; TL/TW: 1.29–1.32; PL/PW: 0.51–0.53.

#### Diagnosis

*Coccinellatransversoguttatatransversoguttata* is similar to *C.undecimpunctata* in external structure and colouration, but can be separated from the latter by a diagnostic character; penis-guide in inner view, rhomboidally dilated with apical hastate (Fig. 6E), but in *C.undecimpunctata* penis-guide in inner view, gradually tapering towards its blunt apex.

#### Distribution

Pakistan. Gilgit-Baltistan (Rafi et al. 2005, Ashfaque et al. 2013, current study), Baluchistan (Mohibullah et al. 2019), Khyber Pakhtunkhwa (current study); –China, India, Japan, Kazakhstan, Kyrgyzstan, Mongolia, Nepal, Russia, northern Scandinavia (Poorani 2002, Kovář 2005, Kovář 2007, Poorani 2023).

#### Ecology

**Prey.** Rafi et al. (2005) reported that this species fed on *Aphiscraccivora* Koch, *A.fabae* Scopoli, *A.gossypii* Glover, *Myzuspersicae* (Sulzer), *Lipaphispseudobrassicae* Davis, *Macrosiphumrosae* (Linnaeus), *Rhopalosiphummaidis* (Fitch), *Therioaphistrifolii* (Monell) and *Pterochloroidespersicae* Cholodkovsky (Hemiptera, Aphididae).

In the current study, this species has been recorded from the following aphid species: *Acyrthosiphonpisum* Harris, *Lachnuswichmanni* Hille Ris Lambers, *Macrosiphumpachysiphon* Hille Ris Lambers, *Aphiscraccivora* Koch, *A.fabae* Scopoli, *A.nerii* Fonscolombe, *A.farinose* (Gmelin), *A.gossypii* Glover, *Schizaphisgraminum* (Rondani), *Tuberolachnussalignus* Gmelin and *Toxopteraaurantii* (Boyer de Fonscolombe) (Hemiptera, Aphididae).

**Associated Plants.** It is collected from the following plants: *Erigeroncanadensis* (Linnaeus) (Asterales, Asteraceae), *Capparisspinosa* Linnaeus (Brassicales, Capparaceae), *Convolvulusarvensis* Linnaeus (Solanales, Convolvulaceae), *Hippophaerhamnoides* Linnaeus (Rosales, Elaeagnaceae), *Salixalba* Linnaeus (Malpighiales, Salicaceae), *Sophoraalopecuroides* Linnaeus (Fabales, Fabaceae), *Rumexdentatus* (Linnaeus) (Caryophyllales, Polygonaceae), *Phragmiteskarka* (Retzius), *Triticumaestivum* Linnaeus (Poales, Poaceae) and *Rosaindica* Trattinnick (Rosales, Rosaceae).

#### Notes

See Kovář (2005) for a detailed description and illustration.

### Coccinella undecimpunctata

Linnaeus, 1758

D1FDF5B0-4384-5DE4-9856-DBB0BB8D518F

Coccinella undecimpunctata Linnaeus, 1758: 366; 89; Mulsant (1866): 85; Iablokoff-Khnzorian (1979): 66; Pope (1989): 651; Poorani (2002);Rafi et al. (2005): 48; Barševskis and Lazdāns (2010); Ali et al. (2012): 169; Hayat et al. (2017): 26; Poorani (2023): 91.
Coccinella aegyptiaca Reiche, 1861: 212: Mulsant (1866): 86.

#### Materials

- **Type status:** Other material. **Occurrence:** individualCount: 5; sex: 1 male, 4 females; occurrenceID: F0EA444B-478B-5D49-83C6-172B329090C1; **Taxon:** scientificName: *Coccinellaundecimpunctata*; order: Coleoptera; family: Coccinellidae; scientificNameAuthorship: Linnaeus 1758; **Location:** country: Pakistan; stateProvince: Punjab; locality: Attock, Por Maina; verbatimElevation: 508 m; decimalLatitude: 33.82764; decimalLongitude: 72.78734; **Event:** year: 2017; month: 5; day: 23; **Record Level:** institutionID: PMAS-AAUR
- **Type status:** Other material. **Occurrence:** individualCount: 4; sex: 4 females; occurrenceID: FABB5CE1-B1A6-53D3-8E11-268AB889AF22; **Taxon:** scientificName: *Coccinellaundecimpunctata*; order: Coleoptera; family: Coccinellidae; scientificNameAuthorship: Linnaeus 1758; **Location:** country: Pakistan; stateProvince: Azad Jammu and Kashmir; locality: Poonch, Rawalakot; verbatimElevation: 1635 m; decimalLatitude: 33.85148; decimalLongitude: 73.77405; **Event:** year: 2017; month: 9; day: 13; **Record Level:** institutionID: PMAS-AAUR

#### Description

**Figure 7.**
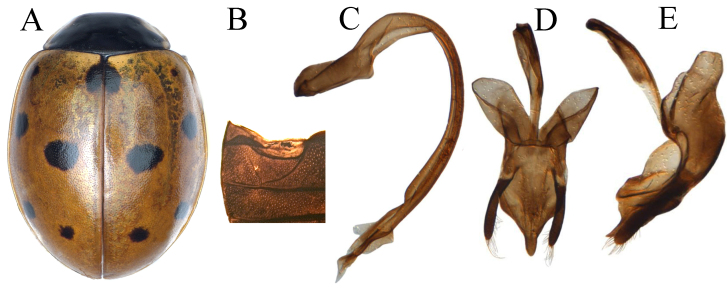
Coccinella (Spilota) undecimpunctata Linnaeus: **A** dorsal view; **B** abdomen; **C** penis; **D** tegmen inner view; **E** tegmen lateral view.

Body (Fig. 7A) broadly oval, convex and dorsal surface glabrous; head black with two yellowish spots at frons, close to the eyes, mouthparts and antennae dark-brown or black; scutellum-black; pronotum black with pale-yellowish elongate marking at anterolateral margins; elytra orange-brown, with five black spots and one common scutellar black spot on each elytron, arranged as 1-2-2, spots round or oval in shape and near to suture relative larger than those of lateral sides; propleura and mesepimera tip white at anterolateral corners; metepimera black with whitish stripe; Male genitalia as in Fig. 7C-E.

##### Measurements

TL: 4.56–4.91 mm; TW: 3.48–3.67 mm; HW: 1.02–1.27 mm; TH: 1.60–1.84 mm; EL/TW: 1.16–1.19; TL/TW: 1.31–1.34; PL/PW: 0.42–0.45; PW/EW: 0.64.

#### Diagnosis

*Coccinellaundecimpunctata* is similar to *C.transversoguttatatransversoguttata* in general body structure and colouration, but can be separated by having a penis guide gradually tapering towards a blunt apex in inner view (Fig. 7D).

#### Distribution

Pakistan. Azad Jammu and Kashmir (Hayat et al. 2017), Baluchistan (Mohibullah et al. 2019), Khyber Pakhtunkhwa (Irshad 2001, Rafi et al. 2005, Saeed et al. 2016), Punjab (Rafi et al. 2005, current study), Sindh (Ali et al. 2012); – Austria, Australia, Azores, Balearic, Belgium, Bulgaria, Denmark, China, Corsica, Czech Republic, England, Finland, France, Germany, Greek, Hungary, India, Iran, Italy, Luxemburg, Mongolia, Nepal, Netherlands, Norway, North Africa, North America, Poland, Portuguese, Sardinia, Slovakia, Spain, Sweden (Poorani 2002, Kovář 2007, Jafari et al. 2015, Poorani 2023).

#### Ecology

**Prey.** Irshad (2001) and Rafi et al. (2005) recorded its feeding on *Aphisgossypii* Glover, *A.malvae*, *A.nerii* Fonscolombe, *Myzuspersicae* (Sulzer) (Hemiptera, Aphididae), eggs of *Pyrillaperpusilla* (Hemiptera, Lophopidae) and *Coccusmangiferae* (Hemiptera, Coccidae). During the present study, *Coccinellaundecimpunctata* feeding was reported on *Brevicorynebrassicae* (Hemiptera, Aphididae).

**Associated Plants.** It was collected from *Brassicaoleracea* (Brassicales, Brassicaceae) during the current study.

## Identification Keys

### Key to species of the genus *Coccinella* from Pakistan

**Table:** 

1	Elytral suture entirely or partially black	2
–	Elytral suture not black	4
2	Anterior half of elytral suture black	*C.luteopicta*
–	Elytral suture entirely black	3
3	Elytral scutellar macula large, broadly rounded (Fig. 3A); prothoracic hypomeron pale yellow at anterior corners; penis apex robust (Fig. 3D); penis guide in lateral view, laterally deeply incised forming apical sub-triangular shape (Fig. 3F)	*C.marussii*
–	Elytral scutellar macula smaller, more elongate oval (Fig. 4A); prothoracic hypomeron white at anterior corners; penis apex blunts with thread-like appendage (Fig. 4C); penis guide in lateral view, laterally entire, regularly converging towards apex (Fig. 4F)	*C.transversalis*
4	Pronotum with brownish sub-quadrangular markings at anterior corners	*C.iranica*
–	Pronotum with white-yellow quadrangular markings at anterior corners	5
5	Elytra with seven black spots or sometimes spots merge and form variable black marks (Fig. 5A-D); pronotal angular spots not connected at anterior margins	*C.septempunctata*
–	Elytra with 11 black spots; pronotal angular spots connected at anterior margins	6
6	Elytral sutural spot sub-triangular (Fig. 6A); metepimera dark brown with whitish spot; penis guide in lateral view, laterally incised forming apical sub-triangular shape (Fig. 6F)	*C.transversoguttata*
–	Elytral sutura spot oval or rounded (Fig. 7A); metepimera black with whitish stop; penis guide in lateral view, laterally entire, regularly converging towards apex (Fig. 7E)	*C.undecimpunctata*

## Analysis

### DNA Barcoding Analysis

**Figure 8.**
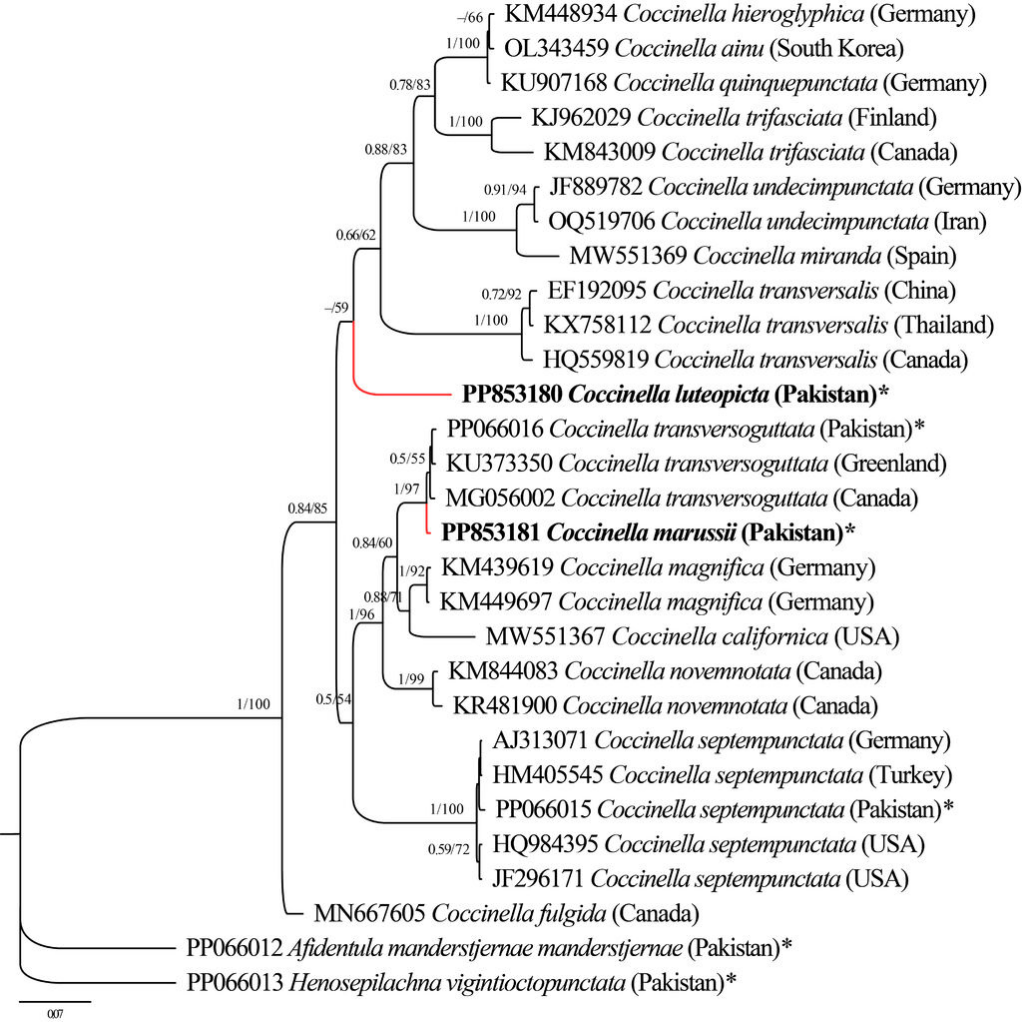
Phylogenetic (BI and ML) tree of *Coccinella* species, based on COI sequences. The numbers displayed on branches represent the Bayesian posterior probability values (left) and bootstrap values for Maximum Likelihood analysis (right). Only branches with bootstrap values above 50% are shown. Sequenced obtained in this study are denoted by an asterisk (*) and newly-submitted sequences to NCBI are highlighted in red and bold.

COI sequences of *C.luteopicta* (Mulsant) and *C.marussii* Kapur were obtained and subsequently submitted to Genbank for the first time, with accession numbers PP853180 and PP853181, respectively. The phylogenetic tree was reconstructed based on the COI barcoding sequences using the Maximum Likelihood (ML) and Bayesian Inference (BI) methods (Fig. 8). Both analyses (ML and BI) yielded the same tree topology, with slight differences in bootstrap (BP) and posterior probability (PP) values. Supporting values for several nodes were weakly to moderately supported, which is expected as only a short fragment of the COI sequence as used. The tree was rooted with the outgroups *Afidentulamanderstjernae* and *Henosepilachnavigintioctopunctata*.

Taxa used in this study are grouped in three clades. The first one includes single species *Coccinellafulgida* with moderate supporting values (PP 0.84 and 85). The second consists of sequences representing six species, *C.transversoguttata*, newly-sequenced *C.marussii*, *C.magnifica*, C. *californica*, *C.novemnotata* and *C.septempunctata*. The last clade contains sequences representing *C.quinquepunctata*, *C.hieroglyphica*, *C.ainu*, *C.trifasciata*, *C.undecimpuntata*, *C.miranda*, *C.transversalis* and newly-obtained sequence of *C.luteopicta* from Pakistan as the most basal within that clade.

## Discussion

The ladybird beetle species was most extensively studied as a potential biocontrol agent against various soft-sucking insect pests in Pakistan. *Coccinellaseptempunctata* Linnaeus is the only representative of this genus with a wide distribution and an extensive prey list, associated with a diverse host plants in Pakistan. In contrast, the three species, *C.luteopicta* (Mulsant), *C.marussii* Kapur and *C.transversoguttata* Faldermann are restricted to the northern regions of Pakistan. This faunistic study is unique compared to the works of Ali et al. (2012), Ashfaque et al. (2013) and Ali et al. (2018) due to broad sampling in various regions of the country which allowed us to find a new country records of *Coccinella* for the Pakistani, *C.luteopicta* (Mulsant, 1866).

*C.septempunctata* is a well known polyphagous species recorded to prey on a wide range of soft insects mainly aphids which was also confirmed for the Pakistani populations (Rafi et al. 2005, Ali et al. 2012, Ashfaque et al. 2013, Saeed et al. 2016). In the current study, it was recorded from more than 60 species of soft-insect pests. On the other hand, *C.luteopicta* and *C.marussii* are oligophagous species feeding on few species of Aphididae.

**Figure 9.**
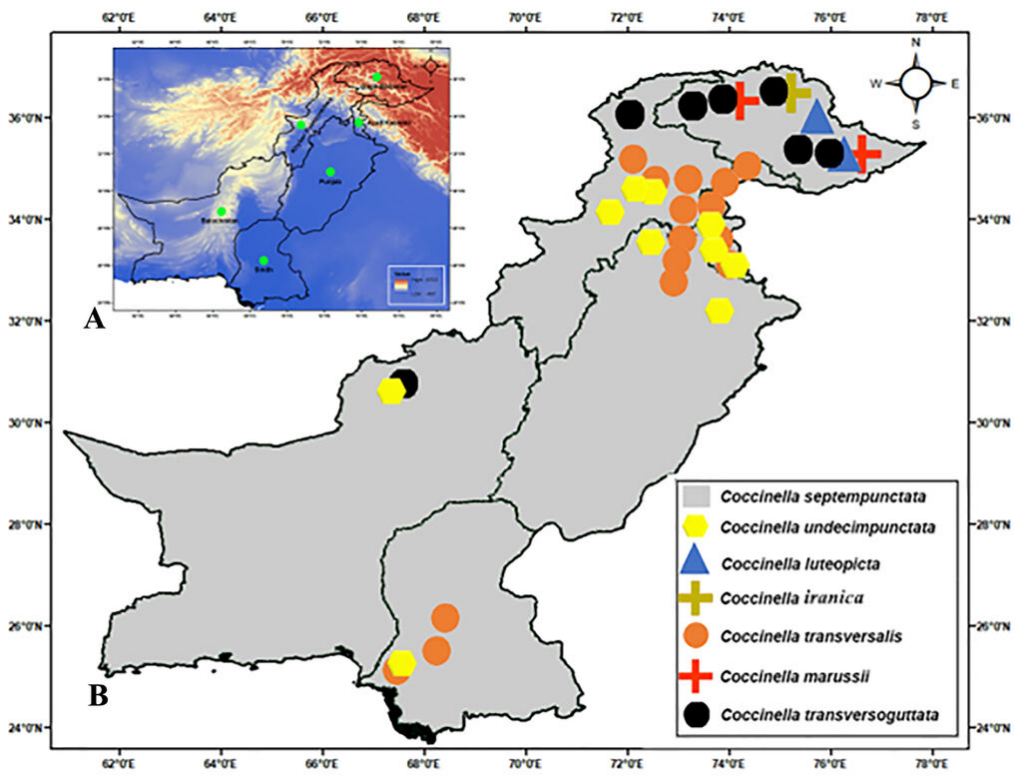
Distribution Map of *Coccinella* species in Pakistan. **A** showing the administrative regions in Pakistan; **B** regional distribution of *Coccinella* species in Pakistan.

Amongst the seven species of Coccinella recorded from Pakistan, four species are found in both Gilgit-Baltistan and Khyber Pakhtunkhwa Provinces, while Azad Kashmir, Balochistan, Punjab and Sindh each host three species (Fig. 9B). This study also expands the known distribution, associated plants and prey lists for all previously recorded and newly-identified species. Based on current sampling, additional studies are recommended to investigate the remaining species, which are uniquely distributed in either the eastern or southern parts of the country. Future research on their geographical distribution, host range and prey will enhance their potential application in Integrated Pest Management in Pakistan.

The analysis of newly-obtained partial COI sequences for *C.luteopicta* and *C.marussii* from Pakistan, within a broader context of other *Coccinella* species from around the world, confirmed their status as distinct species within the genus *Coccinella*. This analysis also suggested possible hypotheses regarding their placement in groups of species, though with weak statistical support. However, previous studies by Milankov et al. (2008) and Magro et al. (2020) have noted that COI gene sequences often fail to differentiate species groups that are morphologically well-defined. As a result, morphologically distinct species frequently share identical COI haplotypes (Mengual et al. 2006). The observed conflict and incongruence between morphological and molecular data emphasise the need for future studies on this genus, employing additional molecular markers.

## Supplementary Material

XML Treatment for Coccinella

XML Treatment for Coccinella luteopicta

XML Treatment for Coccinella marussii

XML Treatment for Coccinella transversalis

XML Treatment for Coccinella iranica

XML Treatment for Coccinella septempunctata

XML Treatment for Coccinella transversoguttata transversoguttata

XML Treatment for Coccinella undecimpunctata

A04417BA-C895-5B1F-91F1-AABD3065F8BC 10.3897/BDJ.12.e137417.suppl1 Supplementary material 1 Table S1 Data type Table Brief description The COI sequences of genus *Coccinella* species used in this study with their GenBank accession numbers. File: oo_1164675.docx https://binary.pensoft.net/file/1164675 Zafar Iqbal, Rashid Azad, Xu Jin, Muhammad Farooq Nasir, Imran Bodlah, Munawar Abbas, Muhammad Ali, Muhammad Asghar Hassan, Karol Szawaryn, Rui-E Nie
