# Supplementary material for: The review of the genus Coccinella (Coleoptera, Coccinellidae) from Pakistan
Source: Biodivers Data J. 2024 Nov 21;12:e137417. doi: 10.3897/BDJ.12.e137417 (PMC11605299; doi:10.3897/BDJ.12.e137417)
Supplement: Supplementary material 1 — Table S1 [file bdj-12-e137417-s001.docx]

**Table S1.** The COI sequences of genus *Coccinella* species used in this study with their GenBank accession numbers.

| **S.#** | **Subfamily** | **Tribe** | **Species** | **Collection location** | **Length (bp)** | **Accession #** | **References** |
| --- | --- | --- | --- | --- | --- | --- | --- |
| 1 | Coccinellinae | Coccinellini | *Coccinella transversalis* | China | 471 | EF192095 | Jing and Yingchun (2006) |
| 2 |  |  | *Coccinella transversalis* | Canada | 658 | HQ559819 | Unpublished |
| 3 |  |  | *Coccinella transversalis* | Thailand | 675 | KX758112 | Unpublished |
| 4 |  |  | *Coccinella transversoguttata* | Greenland | 658 | KU373350 | Writa et al. (2016) |
| 5 |  |  | *Coccinella transversoguttata* | Canada | 588 | MG056002 | Unpublished |
| 6 |  |  | *Coccinella transversoguttata transversoguttata* | Pakistan | 418 | PP066016 | This study |
| 7 |  |  | *Coccinella trifasciata* | Finland | 658 | KJ962029 | Pentinsaari et al. (2014) |
| 8 |  |  | *Coccinella trifasciata* | Canada | 588 | KM843009 | Unpublished |
| 9 |  |  | *Coccinella undecimpunctata* | Germany | 658 | JF889782 | Unpublished |
| 10 |  |  | *Coccinella undecimpunctata* | Iran | 682 | OQ519706 | Unpublished |
| 11 |  |  | *Coccinella ainu* | South Korea | 658 | OL343459 | Unpublished |
| 12 |  |  | *Coccinella californica* | USA | 676 | MW551367 | Nattier et al. (2021) |
| 13 |  |  | *Coccinella fulgida* | Canada | 654 | MN667605 | Pentinsaari et al. (2020) |
| 14 |  |  | *Coccinella hieroglyphica* | Germany | 658 | KM448934 | Hendrich et al. (2015) |
| 15 |  |  | *Coccinella magnifica* | Germany | 658 | KM439619 | Hendrich et al. (2015) |
| 16 |  |  | *Coccinella magnifica* | Germany | 658 | KM449697 | Hendrich et al. (2015) |
| 17 |  |  | *Coccinella miranda* | Spain | 692 | MW551369 | Nattier et al. (2021) |
| 18 |  |  | *Coccinella novemnotata* | Canada | 658 | KM844083 | Unpublished |
| 19 |  |  | *Coccinella novemnotata* | Canada | 591 | KR481900 | Hebert et al. (2016) |
| 20 |  |  | *Coccinella quinquepunctata* | Germany | 658 | KU907168 | Rulik et al. (2017) |
| 21 |  |  | *Coccinella septempunctata* | Germany | 610 | AJ313071 | Hinrich et al. (2002) |
| 22 |  |  | *Coccinella septempunctata* | Turkey | 658 | HM405545 | Unpublished |
| 23 |  |  | *Coccinella septempunctata* | USA | 658 | HQ984395 | Unpublished |
| 25 |  |  | *Coccinella septempunctata* | USA | 658 | JF296171 | Greenstone et al. (2011) |
| 25 |  |  | *Coccinella septempunctata* | Pakistan | 418 | PP066015 | This study |
| 26 |  |  | *Coccinella luteopicta* | Pakistan | 418 | PP853180 | This study |
| 27 |  |  | *Coccinella_marussii* | Pakistan | 418 | PP853181 | This study |
| 28 |  | Epilachnini | *Afidentula manderstjernae* | Pakistan | 418 | PP066012 | This study |
| 29 |  |  | *Henosepilachna vigintioctopunctata* | Pakistan | 418 | PP066013 | This study |
